# Illness perceptions, fear of progression and health-related quality of life during acute treatment and follow-up care in paediatric cancer patients and their parents: a cross-sectional study

**DOI:** 10.1186/s40359-023-01078-6

**Published:** 2023-02-13

**Authors:** Kristina Herzog, Florian Schepper, Thomas Pletschko, Jessy Herrmann, Mihaela Budich, Holger Christiansen, Meinolf Suttorp, Julia Martini

**Affiliations:** 1 Department of Psychiatry and Psychotherapy, Faculty of Medicine of the Technische Universität Dresden, Fetscherstr. 74, 01307 Dresden, Germany; 2 Department of Paediatric Oncology, Haematology and Haemostaseology, Leipzig University, Leipzig, Germany; 3 Department of Paediatric and Adolescent Medicine, Medical University Vienna, Vienna, Austria; 4 Elternhilfe für krebskranke Kinder e.V. Leipzig, Leipzig, Germany; 5 Department of Paediatrics, Paediatric Haematology and Oncology, University Hospital Carl Gustav Carus Dresden, Dresden, Germany; 6 Faculty of Medicine, Technische Universität Dresden, Dresden, Germany

**Keywords:** Cancer, Cancer survivors, Disease progression, Fear, Health-related quality of life, Oncology, Paediatric, Parents, Psycho-oncology, Psychosocial support

## Abstract

**Background:**

This study examines the role of illness perceptions and fear of progression (FoP) in paediatric cancer patients and their parents for patient’s health-related quality of life (HRQoL), controlling for sociodemographic and medical variables. 4–18-year-old patients in acute treatment or follow-up care and one parent were examined.

**Methods:**

*N* = 46 patient-parent dyads in acute treatment and *n* = 84 dyads in follow-up care completed measures on illness perceptions (Illness-Perceptions-Questionnaire for 12–18-year-old patients and parents or as age-adapted puppet interview for 4–11-year-old patients) and FoP (Fear-of-Progression-Questionnaire for 7–18-year-old patients and parents). Patients also completed the KINDL-R to measure HRQoL. Hierarchical multiple regression analyses were calculated.

**Results:**

In acute treatment, patient’s perceptions of symptoms and cyclicity of their illness explained variation in their HRQoL in addition to sociodemographic and medical variables. In follow-up care, patient’s FoP and parent’s perception of consequences explained additional variation in patient’s HRQoL. Overall, sociodemographic and medical variables explained less variation in HRQoL in follow-up care than in acute treatment.

**Conclusions:**

Our results stress the importance of psychological factors for the well-being of paediatric cancer patients, particularly in follow-up care, where sociodemographic and medical variables play a lesser role. We recommend screening for illness perceptions and FoP during and after acute treatment to support patients and parents. Furthermore, standardized interventions focussed on changing maladaptive illness perceptions should be developed and evaluated. As parents’ perceptions, thoughts, and feelings may also play an important role for the well-being of the patients, interventions should be family-focussed and include parents.

*Trial registration* The study has been pre-registered at the German Clinical Trials Register (registered 30/06/2020; DRKS00022034) and at the Open Science Framework (https://osf.io/3uwrx).

**Supplementary Information:**

The online version contains supplementary material available at 10.1186/s40359-023-01078-6.

## Background

Every year, approximately one in 400 children in Germany is diagnosed with cancer [1]. In the last decades, treatment options have improved significantly, leading to increased survival rates of 82% for any cancer diagnosis, and even 90% for acute lymphoblastic leukaemia [1]. Despite improved prognosis, many paediatric patients and their families report poor health-related quality of life (HRQoL) both during and after acute treatment [2–7], that cannot be explained completely by medical variables such as diagnosis or treatment.

Cognitive variables may be associated with HRQoL and are crucial to be considered in psychosocial interventions. Among such variables are individual perceptions and emotions associated with the illness: According to the Common-Sense Model of Illness Representation (CSM) [8], these so-called *illness perceptions* are cognitive representations of a perceived health threat, e.g., a cancer diagnosis. They are based on available information (e.g., conversations with others, information by medical staff, own experience) and launch a self-regulation process which aims to overcome the health threat. Illness perceptions comprise several dimensions: Perceptions on physical symptoms associated with the illness, expected timeline, causes, consequences, controllability and curability, and illness-related emotions [9]. Recent evidence suggests that illness perceptions explain a substantial proportion of variance in HRQoL in both adult and adolescent patients with chronic illness [10–14]. This effect has also been shown in adult cancer survivors [15–17], indicating that the association between illness perceptions and HRQoL persists even until after acute treatment.

Another important variable that has been shown to be associated with HRQoL is fear of progression (FoP). FoP is among the most commonly experienced psychological burdens in acute treatment and follow-up care for adult cancer patients [18] and is defined as the fear that the illness will progress, spread, or recur [19]. It is a rational response to a realistic life-threatening illness and therefore has to be distinguished from neurotic anxiety disorders. Nevertheless, FoP may range from functional (e.g., thereby retaining treatment compliance) to dysfunctional levels. Dysfunctional levels have been shown to be associated with lowered HRQoL both in child and adult cancer patients during acute treatment [20, 21], in adult cancer survivors [22, 23], and in parents of childhood cancer survivors [24].

The CSM poses that, apart from personal experience with the illness, patient’s illness perceptions are also shaped through interaction with significant others. Studies on adult cancer patients found that carer’s/spouse’s illness perceptions are significantly associated with patient’s HRQoL, anxiety, or depression [25, 26]. Similar results have been found in adult oesophageal cancer survivors and their carers [16]. Similarly, parental FoP may inhibit interactions between the parent and their cancer-sick child, as well as family functioning through the transference of anxiety, as has been indicated in an expert survey with professionals in paediatric oncology [27].

Research in paediatric oncology has already demonstrated the relevance of sociodemographic and medical variables on illness perceptions, fear of progression, and HRQoL: Child’s age and gender are often identified as important sociodemographic correlates [28–30], while diagnosis, time since diagnosis, and treatment modality have been identified as important medical variables [6, 28, 29, 31].

This paper aims to examine the following research question: To what extent do child’s and parent’s illness perceptions and FoP predict child’s HRQoL in acute treatment and follow-up care, when controlling for sociodemographic and medical variables? We hypothesize that even after controlling for sociodemographic and medical variables, child’s and parent’s illness perceptions and their FoP predict child’s HRQoL in a hierarchical multiple regression analysis. Analyses will be performed separately by treatment stage (acute treatment and follow-up care).

## Methods

### Design and procedure

This is an observational cross-sectional study. Participants in acute treatment were recruited in the acute wards for paediatric oncology at the university hospitals in Dresden and Leipzig, Germany. Participants in follow-up care were recruited in the parents’ associations in Dresden and Leipzig (Sonnenstrahl e.V. Dresden, Elternhilfe e.V. Leipzig). One parent was also invited to participate. Inclusion criteria were (1) cancer patients aged 4–18 years and one parent, (2) in acute treatment: first diagnosis ≥ 1 month ago, (3) in follow-up care: first diagnosis ≥ 2 years ago; being off active treatment. Exclusion criteria were (1) inability to understand questionnaire/puppet interview, (2) lack of German language knowledge, (3) palliative care.

Children and parents completed measures of HRQoL, illness perceptions, and FoP. Information on sociodemographic and medical characteristics of the child was obtained from the parent.

The study has been pre-registered at the German Clinical Trials Register (registered 30/06/2020; DRKS00022034) and at the Open Science Framework (https://osf.io/3uwrx).

### Participants

**Fig. 1:**
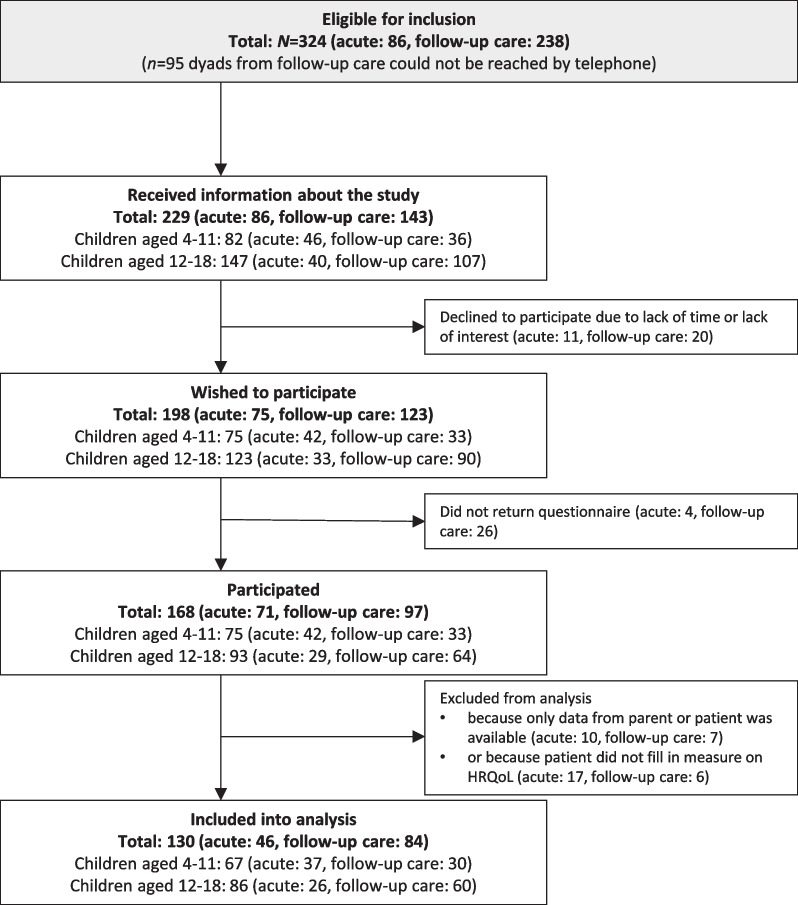
Flow chart on participation

Overall, *n* = 229 families were eligible for inclusion and informed by our staff about the study. Of those, *n* = 198 (86%) families wished to participate (Fig. 1). To be included into analysis, data from both child (including the dependent variable child’s HRQoL) and parent had to be available.

In total,* n* = 46 child-parent dyads in acute treatment and *n* = 84 child-parent dyads in follow-up care were available for this study. Sociodemographic and illness characteristics are summarized in Table 1.

**Table 1:** Sample characteristics

	Acute treatment (*n* = 46)	Follow-up care (*n* = 84)
*Children*		
Gender (*n*, %)		
Male	17 (37.0)	53 (63.1)
Female	29 (63.0)	31 (36.9)
Diverse	0 (0.0)	0 (0.0)
Age (*M, SD*)	10.6 (3.8)	12.6 (3.3)
Diagnosis (*n*, %)		
Leukaemia	15 (32.6)	32 (38.1)
Lymphoma	7 (15.2)	10 (11.9)
Tumour of the central nervous system	8 (17.4)	24 (28.6)
Other solid tumour^†^	16 (34.8)	18 (21.4)
Time since diagnosis in months (*M, SD*)	7.6 (17.4)	79.7 (44.0)
Treatment modality (*n*, %—*multiple responses possible)*		
Chemotherapy	41 (89.1)	73 (86.9)
Radiotherapy	7 (15.2)	21 (25.0)
Surgical measures	21 (45.7)	37 (44.0)
Haematopoietic stem cell transplant	1 (2.2)	8 (9.5)
Other^‡^	0 (0.0)	5 (5.9)
Combination of treatment modalities		
Unimodal	27 (58.7)	46 (54.8)
Multimodal (more than one modality)	19 (41.3)	38 (45.2)
*Parent*		
Participating parent (*n*, %)		
Mother	40 (87.0)	69 (82.1)
Father	6 (13.0)	15 (17.9)
Age (*M, SD*)	38.3 (5.8)	42.7 (6.1)

^†^e.g., Ewing sarcoma, Hepatoblastoma

^‡^e.g., immune therapy, proton beam irradiation

### Measures

#### KINDL-R

The KINDL-R [30] was used to ask all children about their own HRQoL during the past week (e.g., “In the past week, I felt anxious or insecure.”). The questionnaire consists of seven dimensions: (1) Physical well-being, (2) emotional well-being, (3) self-esteem, (4) family, (5) friends, (6) daily functioning (school or pre-school/nursery school), and (7) illness. The items are measured on five-point Likert-scales ranging from 1 (never) to 5 (always), except for the version for 4–6-year-old children (Kiddy-KINDL-R), which consists of a three-point Likert scale. The score for HRQoL is calculated by taking the mean of the items following any reverse scoring. For the present study, all scores were transformed to values between 0 to 100 as specified by the manual: Score_100_ = ([sum score – 24]/96)*100 [32].Higher scores indicate higher HRQoL [30]. The KINDL-R shows acceptable to good internal consistency and high convergent and discriminant validity [33, 34]. In the present study internal consistency for the KINDL-R version for 7–18-year-olds was good (*α* = 0.85), while Cronbach’s Alpha for the Kiddy-KINDL-R could not be calculated due to too much missing data.

#### Illness perception questionnaire–revised (IPQ-R)

Illness perceptions of children and parents were assessed using the German IPQ-R [35], which consists of seven dimensions, relative to the CSM: Identity (symptom attribution), timeline-acute/chronic (duration), timeline-cyclical (perceived trajectory as constant or cyclical), personal control (patient’s influence over the illness), illness coherence (comprehension of the illness), consequences (impact of the illness on their life), and emotional representations (emotional impact). The IPQ-R assesses the symptom-dimension with 14 dichotomous items (yes/no) and the other dimensions with 18 items (3 items per dimension) on a five-point Likert-scale ranging from 1 (strongly disagree) to 5 (strongly agree). The dimension scores are calculated by summing the items after reverse scoring [36]. Higher scores indicate more associated symptoms, more negative perception of chronicity, cyclicity, and emotional representations, and more positive perception of personal control and illness coherence.

Parents and 12–18-year-old children completed the IPQ-R questionnaire. Parents were asked about their own perceptions regarding their child’s illness (e.g., “I get depressed when I think about my child’s illness”). 12–18-year-olds were given a paper–pencil questionnaire similar to the parent’s version (e.g., “I get depressed when I think about my illness”). 4–11-year-old children were examined using an age-adapted hand puppet interview containing the IPQ-R questions [37, 38]. This allowed us to investigate illness perceptions in children and their parents using the same items. For the puppet interview, the IPQ-R items have been dichotomized and two hand puppets tell the child about their opposing views of their own illness (e.g., “I am sad because of my illness.” vs. “I am not sad…”). The child then decides which puppet’s view corresponds with his/her own perception. It could be argued that the questionnaire and the puppet interview do not measure the exact same construct. However, the puppet interview is a state-of-the-art approach to conduct assessments in young children [39], and the items of the IPQ-R were carefully age-adapted, retaining their meaning. The puppet interview showed acceptable validity, comprehensibility, and feasibility in two samples of *n* = 11 children (pilot study) and *n* = 64 children in acute treatment and follow-up care [37, 38].

For the present analysis, scores for the 12–18-year-old children’s IPQ-R were dichotomized (except symptoms-dimension; 0 = scores 1 [strongly disagree] to 3 [neither disagree nor agree]; 1 = scores 4 [agree] and 5 [strongly agree]) to match the scale format of the IPQ-R-Puppet scores, yielding a consistent IPQ-R scale for all children. Previous studies showed sufficient internal consistency (except timeline-cyclical) [35], test–retest-reliability (except timeline-cyclical) [36], and construct validity [38] of the IPQ-R. In the present study, internal consistency for the parental IPQ-R dimensions except timeline-cyclical and coherence was acceptable [40] (Cronbach’s alpha: *α*_*timeline-acute/chronic*_ = 0.715, *α*_*timeline cyclical*_ = 0.454, *α*_*consequences*_ = 0.732, *α*_*personal control*_ = 0.680, *α*_*coherence*_ = 0.606, *α*_*emotional representations*_ = 0.794). Internal consistency of child’s dichotomous IPQ-R dimensions was acceptable for the dimensions timeline-acute/chronic and emotional representations (Kuder-Richardson-20 formula: *α*_*timeline-acute/chronic*_ = 0.707, *α*_*timeline cyclical*_ = 0.244, *α*_*consequences*_ = 0.450, *α*_*personal control*_ = 0.543, *α*_*coherence*_ = 0.618, *α*_*emotional representations*_ = 0.656).

#### Fear of progression questionnaire–revised (FoP-Q)

All parents completed the German adaptation of the FoP-Q-SF for parents (FoP-Q-SF/P) [41]. Children from the age of 7 years completed the adaption for children (FoP-Q-SF/C) [20]. The FoP-Q measures FoP with 12 items on a five-point Likert-scale ranging from 1 (never) to 5 (very often). Children and parents were asked about their own FoP, respectively (e.g., “I become anxious when I think that my child's disease may progress.” vs. “I become anxious when I think that my illness may progress.”). The FoP score is calculated by summing the items. Higher scores indicate greater FoP. The FoP-Q-SF/P and FoP-Q-SF/C showed good internal consistency, convergent and discriminant validity [20, 41]. In the present study internal consistency for the FoP-Q-SF/P (*α* = 0.90) and FoP-Q-SF/C (*α* = 0.88) was good.

### Statistical analysis

Analyses were performed using IBM SPSS Statistics 27.0 [42].

#### Imputation of missing values for independent variables

Across cases in acute treatment, 1.45% of independent variable values (child’s and parent’s illness perceptions, child’s and parent’s FoP) were missing. In 69.57% of cases, all values were available. Across cases in follow-up care, 1.84% of variable values were missing, while all values were available in 78.05% of cases. Little’s test showed that values of the independent variables were completely missing at random (MCAR), *Χ*^*2*^(90) = 87.071, *p* = 0.568. Therefore, missing values for the independent variables were imputed using multiple imputation with *m* = 5 imputations (linear regression model), and the pooled imputed data was used for the analyses described below [43].

#### Associations between dependent and independent variables

Independent *t*-tests, one-way independent ANOVA, Kendall-Tau correlations, and pointbiserial correlations were conducted to investigate associations between sociodemographic (child’s age, gender), medical variables (diagnosis, time since diagnosis, treatment modality), and child’s and parent’s illness perceptions and FoP with the child’s HRQoL. The analyses were run separately by stage of medical treatment (acute treatment, follow-up care).

#### Hierarchical multiple regression analyses

To investigate if child’s and parent’s illness perceptions and FoP predict child’s HRQoL, two hierarchical multiple regression analyses were conducted (separately by stage of treatment). Important sociodemographic and medical variables (child’s age, diagnosis [dummy coded, reference category: leukaemia], time since diagnosis) were entered first to control for their potential influence. In the second step, only those independent variables (illness perceptions, FoP) that were found to be significantly associated with HRQoL were entered.

Assumptions for hierarchical multiple regression analyses were examined before interpretation of results: Residuals of the dependent variable (child’s HRQoL) were examined for normal distribution (histogram, Kolmogorov–Smirnov test, Shapiro–Wilk test) and homoscedasticity (White test), and the data was examined for linearity of association between the independent variables and the dependent variable (partial regression diagrams), multicollinearity, and outliers (studentized deleted residuals, Cook’s distances, leverages).

For all tests *p* < 0.05 was considered statistically significant.

## Results

### Descriptives

Descriptive results of IPQ-R, FoP-Q-SF, and KINDL-R, and correlations between them are summarized in the Additional file 1, Additional file 2 and Additional file 3.

### Associations of sociodemographic and medical characteristics with HRQoL

Child’s HRQoL correlated significantly with their age during acute treatment (*r*_*τ*_ = − 0.275, *p* = 0.009) and follow-up care (*r*_*τ*_ = − 0.277, *p* < 0.001), with younger children reporting higher HRQoL. Gender was not significantly associated with HRQoL in acute treatment (*r*_*pb*_ = 0.064, *p* = 0.673) and follow-up care (*r*_*pb*_ = − 0.126, *p* = 0.256).

One-way ANOVAs showed that diagnosis was not significantly associated with HRQoL in patients in acute treatment (*F*(3,42) = 2.357, *p* = 0.085) and follow-up care (*F*(3,80) = 0.164, *p* = 0.920).

Longer time since cancer diagnosis was significantly correlated with lower HRQoL in follow-up care (*r*_*τ*_ = − 0.229, *p* = 0.002), but not in acute treatment (*r*_*τ*_ = 0.174, *p* = 0.107).

Among patients in acute treatment, there was no significant difference in HRQoL between patients receiving only one modality of treatment and those receiving more than one modality of treatment, *t*(44) = − 1.547, *p* = 0.129. In follow-up care, patients who had received only one modality of treatment reported higher HRQoL (*M* = 78.13, *SD* = 11.54) than patients who had received more than one modality of treatment (*M* = 71.88, *SD* = 14.27), *t*(82) = 2.222, *p* = 0.029.

### Associations of illness perceptions and FoP with HRQoL

Table 2 shows correlations between illness perceptions and FoP with HRQoL. In acute treatment, child’s perception of associated symptoms and cyclical trajectory, as well as parent’s perception of associated symptoms and chronicity were significantly associated with HRQoL. In follow-up care, child’s perception of associated symptoms, parent’s perception of consequences, and child’s FoP were significantly associated with HRQoL.

**Table 2:** Kendall-Tau correlations *(r*_*τ*_*, p)* of illness perceptions and FoP with HRQoL, by stage of medical treatment

	Health related quality of life (HRQoL), by stage of treatment	
	Acute treatment (*n* = 46)	Follow-up care (*n* = 84)
Child’s illness perceptions		
IPQ-R Symptoms	** − .302 (.005)**	** − .254 (.021)**
IPQ-R Timeline-acute/chronic	.103 (.368)	.037 (.738)
IPQ-R Timeline-cyclical	** − .308 (.008)**	− .126 (.260)
IPQ-R Consequences	− .213 (.064)	− .204 (.064)
IPQ-R Coherence	.015 (.895)	.046 (.677)
IPQ-R Personal control	− .033 (.827)	.195 (.077)
IPQ-R Emotional representations	− .006 (.961)	− .095 (.391)
Parent’s illness perceptions		
IPQ-R Symptoms	** − .235 (.029)**	− .150 (.186)
IPQ-R Timeline-acute/chronic	**.269 (.013)**	− .053 (.633)
IPQ-R Timeline-cyclical	.058 (.702)	− .042 (.705)
IPQ-R Consequences	.168 (.118)	** − .219 (.046)**
IPQ-R Coherence	− .080 (.455)	.077 (.486)
IPQ-R Personal control	− .125 (.238)	− .023 (.838)
IPQ-R Emotional representations	.134 (.215)	− .035 (.753)
Child’ FoP	− .108 (.501)	** − .319 (.004)**
Parent’s FoP	.063 (.549)	− .039 (.726)

Bold characters indicate a significant result (*p* < 0.05)

### Hierarchical multiple regression analyses

#### Acute treatment

Table 3 shows regression and determinant coefficients for each step of the hierarchical multiple regression analysis.

**Table 3:** Hierarchical multiple regression analysis for the outcome child’s HRQoL in acute treatment (*n* = 46)^†^

Predictor	*B*	SE *B*	*β*	*t* (*p*)	*R*^*2*^	*ΔR*^*2*^	*F* change (*p*)
*Step 1: Sociodemographic and medical variables*					.401	.401	5.363 (< .001)
(Constant)	79.134	5.046		15.682 (< .001)			
Child’s Age	− 1.292	0.443	− .378	− 2.916 (.006)			
Diagnosis: lymphoma (dummy variable)^‡^	− 2.451	5.013	− .069	− 0.489 (.628)			
Diagnosis: tumour of the central nervous system (dummy variable)^‡^	2.006	4.809	.060	0.417 (.679)			
Diagnosis: other solid tumour (dummy variable)^‡^	7.134	3.914	.267	1.823 (.076)			
Time since diagnosis	0.297	0.095	.402	3.132 (.003)			
*Step 2: Child’s illness perceptions*					.498	.097	3.643 (.036)
(Constant)	86.254	5.664		15.230 (< .001)			
Child’s Age	− 1.033	0.521	− .302	− 1.983 (.055)			
Diagnosis: lymphoma (dummy variable)^‡^	− 3.821	5.048	− .108	− 0.757 (.454)			
Diagnosis: tumour of the central nervous system (dummy variable)^‡^	− 1.810	4.735	− .054	− 0.382 (.704)			
Diagnosis: other solid tumour (dummy variable)^‡^	5.486	3.819	.205	1.436 (.159)			
Time since diagnosis	0.271	0.090	.366	3.012 (.005)			
Child’s IPQ-R Symptoms	− 0.203	0.624	− .057	− 0.325 (.747)			
Child’s IPQ-R Timeline-cyclical	− 4.563	1.953	− .314	− 2.336 (.025)			
*Step 3: Parent’s illness perceptions*					.541	.044	1.718 (.194)
(Constant)	75.807	8.993		8.429 (< .001)			
Child’s Age	− 1.171	0.520	− .342	− 2.253 (.030)			
Diagnosis: lymphoma (dummy variable)^‡^	− 1.729	5.180	− .049	− 0.334 (.740)			
Diagnosis: tumour of the central nervous system (dummy variable)^‡^	− 1.616	4.651	− .048	− 0.348 (.730)			
Diagnosis: other solid tumour (dummy variable)^‡^	5.650	3.830	.211	1.475 (.149)			
Time since diagnosis	0.163	0.106	.220	1.540 (.132)			
Child’s IPQ-R Symptoms	0.744	0.954	.209	0.781 (.440)			
Child’s IPQ-R Timeline-cyclical	− 5.381	1.978	− .371	− 2.721 (.010)			
Parent’s IPQ-R Symptoms	− 0.747	0.823	− .200	− 0.907 (.370)			
Parent’s IPQ-R Timeline-acute/chronic	1.489	0.820	.273	1.816 (.078)			

^†^As no data of the variables included in the hierarchical multiple regression model was missing, the imputed model was the same as the original data set. Therefore, regression coefficients, determination coefficients and the* F*-statistics were the same across all *m* = 5 imputations and the original data set

^‡^Reference category: Leukaemia

All assumptions for a hierarchical multiple regression analysis were met (see Additional file 4).

Admitting sociodemographic and medical variables (child’s age, diagnosis, time since diagnosis) into the model explained 40.1% of variance in child’s HRQoL, *F*(5,40) = 5.363, *p* < 0.001. Introducing child’s perceptions of associated symptoms and cyclicity to the model explained an additional 9.7% of variation in child’s HRQoL. This change in *R*^*2*^ was significant, *F*(2,38) = 3.643, *p* = 0.036. Introducing parent’s perceptions of associated symptoms and chronicity in the third step did not significantly explain additional variation in child’s HRQoL, *ΔR*^*2*^ = 0.064, *F*(2,36) = 1.718, *p* = 0.194. The regression model after step 2 yielded the following regression coefficients: Children with longer intervals from diagnosis to interview reported higher HRQoL (*B* = 0.271), and children with higher perception of cyclical trajectory reported lower HRQoL (*B* = − 4.563).

#### Follow-up care

Table 4 shows regression and determinant coefficients for each step of the hierarchical multiple regression analysis.

**Table 4:** Hierarchical multiple regression analysis for the outcome child’s HRQoL in follow-up care (*n* = 84)

Predictor	*B*	SE *B*	*t* (*p*)	*R*^*2* †^	*ΔR*^*2* †^	*F* change (*p*)^†^
*Step 1: Sociodemographic and medical variables*				.22	.22	4.406 (.001)
(Constant)	99.670	5.826	17.107 (< .001)			
Child’s Age	− 1.432	0.446	− 3.208 (.001)			
Diagnosis: lymphoma (dummy variable)^‡^	− 2.958	4.416	− 0.670 (.503)			
Diagnosis: tumour of the central nervous system (dummy variable)^‡^	− 3.682	3.261	− 1.129 (.259)			
Diagnosis: other solid tumour (dummy variable)^‡^	− 1.865	3.603	− 0.518 (.605)			
Time since diagnosis	− 0.056	0.034	− 1.684 (.092)			
*Step 2: Child’s illness perceptions*				.28	.06	4.886 (< .001)
(Constant)	99.488	5.634	17.660 (< .001)			
Child’s Age	− 0.889	0.484	− 1.838 (.066)			
Diagnosis: lymphoma (dummy variable)^‡^	− 1.890	4.290	− .440 (.660)			
Diagnosis: tumour of the central nervous system (dummy variable)^‡^	− 4.716	3.184	− 1.481 (.139)			
Diagnosis: other solid tumour (dummy variable)^‡^	− 1.684	3.485	− 0.483 (.629)			
Time since diagnosis	− 0.066	0.033	− 2.004 (.045)			
Child’s IPQ-R Symptoms	− 1.104	0.445	− 2.479 (.013)			
*Step 3: Child’s FoP*				.35	.07	5.563 (< .001)
(Constant)	105.506	5.854	18.023 (< .001)			
Child’s Age	− 0.787	.466	− 1.689 (.091)			
Diagnosis: lymphoma (dummy variable)^‡^	− 0.772	4.128	− 0.187 (.852)			
Diagnosis: tumour of the central nervous system (dummy variable)^‡^	− 3.211	3.097	− 1.037 (.300)			
Diagnosis: other solid tumour (dummy variable)^‡^	− 2.478	3.352	− 0.739 (.460)			
Time since diagnosis	− 0.059	0.031	− 1.870 (.062)			
Child’s IPQ-R Symptoms	− 0.875	0.438	− 1.999 (.046)			
Child’s FoP	− 0.394	0.144	− 2.739 (.006)			
*Step 4: Parent’s illness perceptions*				.42	.07	6.343 (< .001)
(Constant)	118.106	7.124	16.579 (< .001)			
Child’s Age	− 0.966	0.448	− 2.155 (.031)			
Diagnosis: lymphoma (dummy variable)^‡^	− 2.293	3.975	− 0.577 (.564)			
Diagnosis: tumour of the central nervous system (dummy variable)^‡^	− 0.581	3.091	− 0.188 (.851)			
Diagnosis: other solid tumour (dummy variable)^‡^	− 2.686	3.205	− 0.838 (.402)			
Time since diagnosis	− 0.040	0.031	− 1.296 (.195)			
Child’s IPQ-R Symptoms	− 0.769	0.415	− 1.853 (.064)			
Child’s FoP	− 0.361	0.140	− 2.585 (.010)			
Parent’s IPQ-R Consequences	− 1.269	0.440	− 2.886 (.004)			

^†^*R*^*2*^ and *ΔR*^*2*^ were computed using the pooling technique suggested by van Ginkel [46]. *F* scores and *p* values were calculated using the miceadds-package from the statistics software *R*. Results from the original, non-imputed data set are comparable to those of the multiply imputed data set and are shown in the Additional file 6

^‡^Reference category: Leukaemia

All assumptions for a hierarchical multiple regression analysis were met (see Additional file 5).

Admitting sociodemographic and medical variables (age, diagnosis, time since diagnosis) into the model explained 22.0% of variance in child’s HRQoL, *F*(5,78) = 4.406, *p* = 0.001. Introducing child’s perceptions of associated symptoms explained an additional 6.0% of variation in child’s HRQoL. This change in *R*^*2*^ was significant, *F*(6,3099.40) = 4.886, *p* < 0.001. Introducing child’s FoP in the third step explained an additional 7.0% of variation, *F*(7,767.49) = 5.563, *p* < 0.001. Introducing parent’s perceptions of consequences (in the last step explained another 7.0% of variation in child’s HRQoL, *F*(7,678.48) = 6.343, *p* < 0.001. The final regression model yielded the following regression coefficients: Younger children reported higher HRQoL (*B* = − 0.966), children with higher FoP reported lower HRQoL (*B* = − 0.361), and children whose parents perceived more negative consequences of the illness reported lower HRQoL (*B* = − 1.269).

## Discussion

Research in the last decades has emphasized the need to investigate the role of psychological constructs such as illness perceptions and fear of progression for HRQoL in patients with chronic illness, as these constructs may contribute to HRQoL in addition to medical factors such as the diagnosis. In paediatric oncology, the cancer diagnosis affects the whole family. Therefore, not only the child’s perceptions and emotions may play a role in their HRQoL, but also those of their caregivers [44].

To the best of our knowledge, this is the first study that investigates the role of child’s and parent’s illness perceptions and fear of progression for child’s HRQoL in paediatric oncological acute treatment and follow-up care. Main results of the study were: (1) In acute treatment, child’s perception of associated symptoms and cyclicity of their illness explained additional variance in child’s HRQoL in addition to sociodemographic and medical variables. (2) In follow-up care, child’s FoP and parent’s perception of consequences explained additional variance in child’s HRQoL. (3) Sociodemographic and medical variables explained less variation of HRQoL in follow-up care, compared to acute treatment.

In our sample in acute treatment, sociodemographic and medical variables explained a total of 40.1% variation in the child’s HRQoL: Specifically, younger age and longer time since diagnosis predicted higher HRQoL in this first step. Adding child’s illness perceptions (associated symptoms, perception of cyclical trajectory) to the model explained an additional 9.7% of variation, leading to a total of 49.8% of explained variation in HRQoL, with longer time since diagnosis and the perception of a less cyclical illness trajectory predicting higher HRQoL. Parental perceptions or child’s and parent’s FoP were not significantly associated with HRQoL and did not play a significant role in the explanation of variation in HRQoL. Our findings are in line with previous research on adult cancer patients [11], adolescents with sickle cell disease [12] or inflammatory bowel disease [10], and children with cancer [13] or spinal muscular atrophy [14]. These studies found that, after controlling for medical and sociodemographic variables, illness perceptions were significant predictors of the patient’s HRQoL. Some of the authors also examined specific illness perception dimensions: Fonseca and colleagues [13] found that the perception of negative consequences was the strongest predictor for HRQoL in 8–12-year-old cancer patients, with the perception of associated symptoms, concerns, coherence, and perceived chronicity also explaining a significant amount of variation. Scharloo and colleagues [11] found that, after controlling for age and comorbidity, increased attention to symptoms was associated with lower self-reported emotional and physical functioning, as well as lower self-reported global health, and perception of a cyclical trajectory was associated with lower self-reported role functioning and cognitive functioning in adult head and neck cancer patients.

In follow-up care, the child’s age explained a total of 22.0% of variation in their HRQoL, with diagnosis and time since diagnosis not being significant predictors of HRQoL. In the next regression steps, child’s perception of associated symptoms, child’s FoP, and parent’s perception of negative consequences each contributed to the variation in HRQoL. In the final model, regression coefficients for the variables age, child’s FoP, and parent’s perception of negative consequences were significant, with younger age, lower FoP in the child, and less negative parental perception of consequences predicting higher HRQoL. Our findings relate to previous research on cancer survivors [15–17]: Specifically, Schoormans and colleagues [15] found that the perception of negative consequences, higher perception of treatment control, higher perception of associated symptoms, and more negative emotional representations were associated with lower HRQoL in the survivor. Dempster and colleagues [16] investigated survivor’s and their carer’s perceptions. After controlling for sociodemographic and medical variables, they found that the survivor’s perceptions of negative consequences, personal control, and coherence, as well as the carer’s perception of consequences and treatment control contributed to the survivor’s anxiety and depression.

Results between the samples in acute treatment and follow-up care differed substantially: Sociodemographic and medical variables in the follow-up care sample explained much less variation in HRQoL compared to the sample in acute treatment (22.0% compared to 40.1%). Concerning psychological factors, in acute treatment only the child’s perception of symptoms was significantly associated with HRQoL, whereas in follow-up care, parental illness perceptions and child’s FoP became important predictors for child’s HRQoL. It seems that during acute treatment, side effects (e.g., nausea, pain) play a major role in the child’s daily life and therefore are vital contributing factors to their HRQoL. In follow-up care, on the other hand, the illness becomes less relevant, whereas psychological factors such as FoP and parent’s illness perceptions become more important to the child’s HRQoL. For example, child’s FoP may not be an important factor in acute treatment because children focus on their daily life in the clinic and not on potential consequences and long-term effects of their illness. Children in follow-up care, on the other hand, may perceive FoP as they survey the health threat retrospectively and have already been confronted with its consequences and long-term effects. Similarly, parental perception of illness consequences predicts their child’s HRQoL in follow-up care only, potentially because children and parents communicate about their perceptions and fears.

### Study limitations

We investigated *n* = 46 child-parent-dyads in acute treatment and *n* = 84 dyads in follow-up care, with the children’s age ranging from 4 to 18 years. To our knowledge, this is the first study that includes children from such a young age both during acute cancer treatment and follow-up care. However, the sample size for the regression analysis in acute treatment was too small, as we included nine predictors into the model. In follow-up care, following the suggestion of Harris [45] to have at least ten cases per predictor, the sample size was sufficient. When interpreting the results from the acute treatment sample, the sample size needs to be kept in mind. Nevertheless, our findings contribute to the field of research and should be explored with sufficient sample sizes in the future.

Internal consistency of some dimensions was low in the IPQ-R puppet interview. Potential reasons might be: (a) The low number of items per dimensions (Cronbach’s alpha gets higher the more items are included) [40], and/ or (b) the dichotomous response format that allows only for two opposite responses (lower interrelatedness of the items [40]). Especially young children might be undecided on particular dimensions because in this age there is no coherent concept for the dimensions. Further studies are needed for replication of the results and to explore potential age effects in response patterns on illness perceptions. Nevertheless, despite low internal consistency of selected dimensions, the use of the IPQ-R as a dichotomous puppet interview is still desirable as it enables the user to compare illness perceptions between young children, adolescents, and parents.

As our study was cross-sectional, it could be argued that illness perceptions or FoP are fostered by lower HRQoL, and not the other way round, as we hypothesized. Our future analyses of the longitudinal data will clarify causal relationships between the variables.

### Clinical implications

Our results stress the importance of psychological factors for the well-being of children suffering from cancer. As medical factors and daily stressors (such as symptom perception) are main influencing factors on HRQoL in acute treatment, this stresses the importance of supportive therapies to improve daily coping and activate individual resources. The importance of psychological factors becomes even more pronounced in follow-up care, where sociodemographic and medical variables play a lesser role. We recommend screening for maladaptive illness perceptions and FoP in children and parents during and after acute treatment as they may influence the child’s HRQoL. This can be done using well-established instruments for the measurement of illness perceptions (IPQ-R as questionnaire or puppet interview for younger children) and FoP (FoP-Q-SF). The association between these factors and the child’s HRQoL imply that interventions focussing on changing maladaptive illness perceptions or high FoP might improve the child’s HRQoL. Effective interventions for adult patients already exist. However, there is a need to develop interventions for paediatric patients. It needs to be kept in mind that in paediatric oncology, not only the child’s perceptions, but also the parent’s perceptions, thoughts, and feelings may play an important role in the well-being of the child. Therefore, interventions should be family-focussed and include parental views and fears concerning their child’s illness.

## Supplementary Information

**Additional file 1**. Descriptive results (*M, SD*) of IPQ-R, FoP-Q-SF, and KINDL-R, by stage of medical treatment.

**Additional file 2**. Correlation matrices of study variables in the acute treatment sample in the original data set and the multiply imputed data set.

**Additional file 3**. Correlation matrices of study variables in the follow-up care sample in the original data set and the multiply imputed data set.

**Additional file 4**. Assumptions for a hierarchical multiple regression analysis for the acute treatment sample (*n*=46).

**Additional file 5**. Assumptions for a hierarchical multiple regression analysis for the follow-up care sample (*n*=84).

**Additional file 6**. Hierarchical multiple regression analysis for the outcome child’s HRQoL in follow-up care, using the original dataset (*n*=68).

## Data Availability

The data and materials that support the findings of this study are available from the corresponding author (KH) upon reasonable request.

## Abbreviations

CSM: Common Sense Model of Illness Representation
DRKS: Deutsches Register Klinischer Studien (German Clinical Trials Register)
FoP: Fear of progression
FoP-Q: Fear of Progression Questionnaire
FoP-Q-SF/P: Fear of progression questionnaire–short form–version for parents
FoP-Q-SF/C: Fear of progression questionnaire–short form–version for children
HRQoL: Health-related quality of life
IPQ-R: Illness perception questionnaire–revised
