# Supplementary material for: Illness perceptions, fear of progression and health-related quality of life during acute treatment and follow-up care in paediatric cancer patients and their parents: a cross-sectional study
Source: BMC Psychol. 2023 Feb 13;11:44. doi: 10.1186/s40359-023-01078-6 (PMC9926758; doi:10.1186/s40359-023-01078-6)
Supplement: Supplementary file 1 — Additional file 1. Descriptive results (M, SD) of IPQ-R, FoP-Q-SF, and KINDL-R, by stage of medical treatment. [file 40359_2023_1078_MOESM1_ESM.docx]

## Additional File 1: Descriptive results *(M, SD)* of IPQ-R, FoP-Q-SF, and KINDL-R, by stage of medical treatment

|  | Acute treatment | |  | Follow-up care | |
| --- | --- | --- | --- | --- | --- |
|  | Child’s score  *(M, SD)* | Parent’s score  *(M, SD)*^a^ |  | Child’s score  *(M, SD)* | Parent’s score  *(M, SD)* |
| IPQ-R^b^ |  |  |  |  |  |
| Sum of symptoms associated with the illness | 5.78 (3.61) | 7.39 (3.45) |  | 5.26 (3.35) | 7.69 (2.95) |
| Timeline-acute/chronic | 1.49 (1.17) | 9.15 (2.36) |  | 1.21 (1.17) | 9.93 (3.10) |
| Timeline-cyclical | 1.54 (0.89) | 9.73 (2.29) |  | 1.13 (0.84) | 9.31 (2.47) |
| Consequences | 1.54 (0.86) | 10.73 (2.81) |  | 1.18 (1.01) | 10.82 (2.95) |
| Coherence | 1.37 (1.05) | 8.98 (2.65) |  | 1.63 (1.14) | 8.70 (2.62) |
| Personal control | 1.61 (1.22) | 9.36 (3.18) |  | 1.31 (0.98) | 8.90 (2.75) |
| Emotional representations | 1.50 (1.15) | 12.07 (2.78) |  | 1.12 (1.13) | 11.47 (2.63) |
| FoP (FoP-Q-SF) | 29.23 (8.03) | 37.00 (8.90) |  | 23.99 (9.40) | 34.55 (9.78) |
| HRQoL (KINDL-R) | 70.15 (12.87) | - |  | 75.30 (13.14) | - |

Note. ^a^ Parent’s and child’s IPQ-R dimension scale formats differ, except for the symptoms-dimension. The parent’s IPQ-R scales (except symptoms-dimension) range from +3 to +12, whereas the child’s IPQ-R scales (except symptoms dimension) range from 0 to +3. ^b^ Higher IPQ-R scores indicate more associated symptoms, more negative perception of chronicity, cyclicity, and emotional representations, and more positive perception of personal control and illness coherence.
