# Supplementary material for: Illness perceptions, fear of progression and health-related quality of life during acute treatment and follow-up care in paediatric cancer patients and their parents: a cross-sectional study
Source: BMC Psychol. 2023 Feb 13;11:44. doi: 10.1186/s40359-023-01078-6 (PMC9926758; doi:10.1186/s40359-023-01078-6)
Supplement: Supplementary file 2 — Additional file 2. Correlation matrices of study variables in the acute treatment sample in the original data set and the multiply imputed data set. [file 40359_2023_1078_MOESM2_ESM.docx]

Additional File 2: Correlation matrices of study variables in the acute treatment sample in the original data set (Table A2-a) and the multiply imputed data set (Table A2-b)

Table A2-a. Correlation matrix (*r_τ_, p*) of study variables in the sub-sample during acute treatment (original dataset)

|  |  | 1 | 2 | 3 | 4 | 5 | 6 | 7 | 8 | 9 | 10 | 11 | 12 | 13 | 14 | 15 | 16 | 17 |
| --- | --- | --- | --- | --- | --- | --- | --- | --- | --- | --- | --- | --- | --- | --- | --- | --- | --- | --- |
| 1 | HRQoL | - |  |  |  |  |  |  |  |  |  |  |  |  |  |  |  |  |
| 2 | Child’s IPQ-R Symptoms | **-.302 (.005)** | - |  |  |  |  |  |  |  |  |  |  |  |  |  |  |  |
| 3 | Child’s IPQ-R Timeline-acute/chronic | .103  (.368) | -.205  (.080) | - |  |  |  |  |  |  |  |  |  |  |  |  |  |  |
| 4 | Child’s IPQ-R Timeline-cyclical | **-.308**  **(.008)** | .229  (.054) | -.038  (.766) | - |  |  |  |  |  |  |  |  |  |  |  |  |  |
| 5 | Child’s IPQ-R Consequences | -.213  (.064) | .021  (.861) | .088  (.488) | **.277**  **(.031)** | - |  |  |  |  |  |  |  |  |  |  |  |  |
| 6 | Child’s IPQ-R Coherence | .015  (.895) | -.056  (.631) | -.005  (.966) | .086  (.501) | .118  (.355) | - |  |  |  |  |  |  |  |  |  |  |  |
| 7 | Child’s IPQ-R Personal control | -.034  (.767) | -.025  (.829) | .222  (.079) | .010  (.939) | **.503**  **(<.001)** | .183  (.149) | - |  |  |  |  |  |  |  |  |  |  |
| 8 | Child’s IPQ-R Emotional representations | -.006  (.961) | .108  (.354) | .130  (.297) | .085  (.500) | .071  (.575) | **-.261**  **(.038)** | .024  (.849) | - |  |  |  |  |  |  |  |  |  |
| 9 | Parent’s IPQ-R Symptoms | **-.235**  **(.029)** | **.697**  **(<.001)** | -.147  (.215) | .131  (.275) | .014  (.909) | -.049  (.682) | -.026  (.828) | .090  (.445) | - |  |  |  |  |  |  |  |  |
| 10 | Parent’s IPQ-R Timeline-acute/chronic | **.269**  **(.013)** | **-.247**  **(.026)** | .404  (.001) | -.032  (.788) | .070  (.562) | -.011  (.926) | .069  (.563) | -.066  (.578) | -.190  (.091) | - |  |  |  |  |  |  |  |
| 11 | Parent’s IPQ-R Timeline-cyclical | .063  (.566) | -.119  (.287) | .127  (.288) | .157  (.196) | .076  (.527) | .065  (.589) | .015  (.902) | .046  (.698) | -.043  (.704) | .152  (.183) | - |  |  |  |  |  |  |
| 12 | Parent’s IPQ-R Consequences | .168  (.118) | -.187  (.090) | .184  (.119) | .032  (.789) | **.321**  **(.007)** | .002  (.984) | .152  (.205) | .198  (.092) | -.060  (.595) | **.349**  **(.002)** | .160  (.159) | - |  |  |  |  |  |
| 13 | Parent’s IPQ-R Coherence | -.080  (.455) | .055  (.616) | .002  (.984) | -.059  (.621) | .107  (.368) | .022  (.854) | -.004  (.975) | .011  (.929) | -.013  (.907) | .169  (.133) | -.041  (.719) | .057  (.608) | - |  |  |  |  |
| 14 | Parent’s IPQ-R Personal control | -.125  (.238) | .140  (.199) | -.167  (.153) | .114  (.336) | .156  (.185) | -.013  (.911) | **.256**  **(.029)** | -.038  (.745) | .067  (.544) | -.094  (.396) | -.079  (.481) | -.069  (.532) | .147  (.181) | - |  |  |  |
| 15 | Parent’s IPQ-R Emotional representations | .134  (.215) | -.101  (.363) | .229  (.054) | -.027  (.820) | .121  (.314) | .017  (.885) | .217  (.070) | .081  (.493) | .021  (.854) | **.253**  **(.025)** | .192  (.092) | .136  (.225) | **-.260**  **(.021)** | **-.248**  **(.025)** | - |  |  |
| 16 | Child’ FoP | -.055  (.663) | -.010  (.938) | -.091  (.506) | .019  (.892) | .047  (.735) | .110  (.419) | -.117  (.402) | .131  (.340) | -.025  (.850) | .000  (1.000) | .103  (.431) | .122  (.345) | -.068  (.594) | .067  (.596) | -.012  (.924) | - |  |
| 17 | Parent’s FoP | .063  (.549) | -.191  (.075) | **.269**  **(.019)** | .167  (.153) | **.301**  **(.009)** | .163  (.158) | **.256**  **(.027)** | .020  (.860) | -.110  (.313) | **.280**  **(.010)** | **.317**  **(.004)** | .207  (.056) | -.045  (.680) | -.010  (.924) | **.565**  **(<.001)** | .153  (.225) | - |

Note. Bold characters indicate a significant result (*p*<0.05).

Table A2-b. Correlation matrix *(r_τ_, p)* of study variables in the sub-sample during acute treatment (multiply imputed dataset)

|  |  | 1 | 2 | 3 | 4 | 5 | 6 | 7 | 8 | 9 | 10 | 11 | 12 | 13 | 14 | 15 | 16 | 17 |
| --- | --- | --- | --- | --- | --- | --- | --- | --- | --- | --- | --- | --- | --- | --- | --- | --- | --- | --- |
| 1 | HRQoL | - |  |  |  |  |  |  |  |  |  |  |  |  |  |  |  |  |
| 2 | Child’s IPQ-R Symptoms | **-.302**  **(.041)** | - |  |  |  |  |  |  |  |  |  |  |  |  |  |  |  |
| 3 | Child’s IPQ-R Timeline-acute/chronic | .103  (.500) | -.205  (.173) | - |  |  |  |  |  |  |  |  |  |  |  |  |  |  |
| 4 | Child’s IPQ-R Timeline-cyclical | **-.308**  **(.037)** | .229  (.126) | -.038  (.804) | - |  |  |  |  |  |  |  |  |  |  |  |  |  |
| 5 | Child’s IPQ-R Consequences | -.213  (.156) | .021  (.892) | .088  (.564) | .277  (.062) | - |  |  |  |  |  |  |  |  |  |  |  |  |
| 6 | Child’s IPQ-R Coherence | .024  (.875) | -.059  (.703) | -.011  (.941) | .087  (.566) | .110  (.470) | - |  |  |  |  |  |  |  |  |  |  |  |
| 7 | Child’s IPQ-R Personal control | -.033  (.827) | -.012  (.936) | .230  (.125) | .024  (.878) | **.502**  **(<.001)** | .183  (.227) | - |  |  |  |  |  |  |  |  |  |  |
| 8 | Child’s IPQ-R Emotional representations | -.006  (.971) | .108  (.476) | .130  (.391) | .085  (.574) | .071  (.642) | -.258  (.083) | .034  (.822) | - |  |  |  |  |  |  |  |  |  |
| 9 | Parent’s IPQ-R Symptoms | -.235  (.116) | **.697**  **(<.001)** | -.147  (.332) | .131  (.387) | .014  (.929) | -.054  (.724) | -.011  (.943) | .090  (.554) | - |  |  |  |  |  |  |  |  |
| 10 | Parent’s IPQ-R Timeline-acute/chronic | .269  (.071) | -.247  (.098) | **.404**  **(.005)** | -.032  (.832) | .070  (.647) | -.011  (.944) | .088  (.563) | -.066  (.665) | -.190  (.208) | - |  |  |  |  |  |  |  |
| 11 | Parent’s IPQ-R Timeline-cyclical | .058  (.702) | -.089  (.561) | .157  (.301) | .189  (.212) | .102  (.501) | .090  (.555) | .033  (.827) | .076  (.616) | -.007  (.964) | .174  (.250) | - |  |  |  |  |  |  |
| 12 | Parent’s IPQ-R Consequences | .168  (.266) | -.187  (.214) | .184  (.222) | .032  (.833) | **.321**  **(.029)** | -.002  (.992) | .161  (.289) | .198  (.187) | -.060  (.696) | **.349**  **(.017)** | .191  (.205) | - |  |  |  |  |  |
| 13 | Parent’s IPQ-R Coherence | -.080  (.598) | .055  (.716) | .002  (.988) | -.059  (.697) | .107  (.480) | .021  (.893) | .009  (.951) | .011  (.945) | -.013  (.932) | .169  (.264) | -.007  (.963) | .057  (.707) | - |  |  |  |  |
| 14 | Parent’s IPQ-R Personal control | -.125  (.410) | .140  (.356) | -.167  (.270) | .114  (.454) | .156  (.303) | -.014  (.928) | .260  (.082) | -.038  (.805) | .067  (.661) | -.094  (.537) | -.048  (.752) | -.069  (.652) | .147  (.332) | - |  |  |  |
| 15 | Parent’s IPQ-R Emotional representations | .134  (.376) | -.101  (.505) | .229  (.126) | -.027  (.857) | .121  (.425) | .012  (.938) | .223  (.138) | .081  (.593) | .021  (.892) | .253  (.090) | .197  (.190) | .136  (.368) | -.260  (.081) | -.248  (.097) | - |  |  |
| 16 | Child’ FoP | -.108  (.501) | .049  (.756) | -.082  (.611) | .108  (.530) | .095  (.556) | .117  (.447) | -.052  (.750) | .151  (.351) | .011  (.942) | -.021  (.893) | .097  (.553) | .009  (.957) | -.037  (.816) | .059  (.739) | .009  (.953) | - |  |
| 17 | Parent’s FoP | .063  (.681) | -.191  (.205) | .269  (.071) | .167  (.269) | **.301**  **(.042)** | .164  (.279) | .255  (.087) | .020  (.894) | -.110  (.471) | .280  (.059) | **.326**  **(.027)** | .207  (.168) | -.045  (.769) | -.010  (.947) | **.565**  **(<.001)** | .162  (.295) | - |

Note. Bold characters indicate a significant result (*p*<0.05).
