# Supplementary material for: Illness perceptions, fear of progression and health-related quality of life during acute treatment and follow-up care in paediatric cancer patients and their parents: a cross-sectional study
Source: BMC Psychol. 2023 Feb 13;11:44. doi: 10.1186/s40359-023-01078-6 (PMC9926758; doi:10.1186/s40359-023-01078-6)
Supplement: Supplementary file 3 — Additional file 3. Correlation matrices of study variables in the follow-up care sample in the original data set and the multiply imputed data set. [file 40359_2023_1078_MOESM3_ESM.docx]

Additional File 3: Correlation matrices of study variables in the follow-up care sample in the original data set (Table A3-a) and the multiply imputed data set (Table A3-b)

Table A3-a. Correlation matrix *(r_τ_, p)* of study variables in the sub-sample during follow-up care (original dataset)

|  |  | 1 | 2 | 3 | 4 | 5 | 6 | 7 | 8 | 9 | 10 | 11 | 12 | 13 | 14 | 15 | 16 | 17 |
| --- | --- | --- | --- | --- | --- | --- | --- | --- | --- | --- | --- | --- | --- | --- | --- | --- | --- | --- |
| 1 | HRQoL | - |  |  |  |  |  |  |  |  |  |  |  |  |  |  |  |  |
| 2 | Child’s IPQ-R Symptoms | **-.281**  **(<.001)** | - |  |  |  |  |  |  |  |  |  |  |  |  |  |  |  |
| 3 | Child’s IPQ-R Timeline-acute/chronic | .029  (.726) | **-.198**  **(.027)** | - |  |  |  |  |  |  |  |  |  |  |  |  |  |  |
| 4 | Child’s IPQ-R Timeline-cyclical | -.132  (.121) | .082  (.365) | **.192**  **(.041)** | - |  |  |  |  |  |  |  |  |  |  |  |  |  |
| 5 | Child’s IPQ-R Consequences | **-.212**  **(.012)** | .090  (.316) | **.195**  **(.035)** | **.249**  **(.008)** | - |  |  |  |  |  |  |  |  |  |  |  |  |
| 6 | Child’s IPQ-R Coherence | .057  (.496) | .016  (.858) | **-.194**  **(.036)** | **-.254**  **(.007)** | -.087  (.344) | - |  |  |  |  |  |  |  |  |  |  |  |
| 7 | Child’s IPQ-R Personal control | **.194**  **(.020)** | -.060  (.499) | .036  (.699) | .054  (.565) | .070  (.448) | -.043  (.639) | - |  |  |  |  |  |  |  |  |  |  |
| 8 | Child’s IPQ-R Emotional representations | -.101  (.229) | -.061  (.494) | .154  (.099) | **.235**  **(.012)** | **.254**  **(.006)** | -.143  (.125) | **.252**  **(.007)** | - |  |  |  |  |  |  |  |  |  |
| 9 | Parent’s IPQ-R Symptoms | -.121  (.131) | **.288**  **(.001)** | -.084  (.346) | .132  (.146) | .021  (.814) | .082  (.359) | -.003  (.975) | -.056  (.528) | - |  |  |  |  |  |  |  |  |
| 10 | Parent’s IPQ-R Timeline-acute/chronic | -.052  (.504) | -.076  (.363) | **.409**  **(<.001)** | .054  (.542) | .095  (.274) | -.037  (.668) | -.023  (.792) | .156  (.074) | -.039  (.637) | - |  |  |  |  |  |  |  |
| 11 | Parent’s IPQ-R Timeline-cyclical | -.041  (.608) | .018  (.834) | **.191**  **(.031)** | **.290**  **(.001)** | .094  (.288) | **-.194**  **(.028)** | -.013  (.879) | .042  (.635) | .125  (.140) | **.177**  **(.030)** | - |  |  |  |  |  |  |
| 12 | Parent’s IPQ-R Consequences | **-.217**  **(.006)** | .024  (.779) | .077  (.379) | .126  (.154) | **.278**  **(.001)** | -.154  (.078) | -.008  (.925) | .036  (.682) | **.218**  **(.009)** | **.221**  **(.006)** | **.327**  **(<.001)** | - |  |  |  |  |  |
| 13 | Parent’s IPQ-R Coherence | .076  (.333) | -.053  (.531) | -.070  (.424) | -.100  (.257) | -.059  (.499) | .119  (.175) | .013  (.882) | .035  (.694) | -.098  (.244) | .009  (.908) | .005  (.948) | -.154  (.059) | - |  |  |  |  |
| 14 | Parent’s IPQ-R Personal control | -.020  (.805) | .096  (.258) | -.019  (.833) | .094  (.293) | .140  (.113) | .068  (.440) | .145  (.099) | .005  (.955) | .084  (.321) | -.110  (.177) | .032  (.699) | .100  (.222) | **.190**  **(.021)** | - |  |  |  |
| 15 | Parent’s IPQ-R Emotional representations | -.038  (.636) | .048  (.573) | .113  (.201) | .100  (.264) | .172  (.051) | -.135  (.125) | -.055  (.532) | .067  (.450) | .048  (.571) | .121  (.142) | **.285**  **(.001)** | **.337**  **(<.001)** | **-.348**  **(<.001)** | -.150  (.070) | - |  |  |
| 16 | Child’ FoP | **-.337**  **(<.001)** | **.204**  **(.019)** | .086  (.341) | **.184**  **(.043)** | .169  (.061) | -.116  (.198) | -.085  (.343) | **.245**  **(.007)** | .041  (.637) | .058  (.493) | **.196**  **(.022)** | .049  (.561) | -.028  (.740) | -.066  (.438) | .138  (.109) | - |  |
| 17 | Parent’s FoP | -.040  (.600) | .071  (.384) | .145  (.086) | .165  (.054) | **.208**  **(.014)** | **-.228**  **(.007)** | .060  (.478) | **.170**  **(.045)** | .063  (.434) | **.253**  **(.001)** | **.291**  **(<.001)** | **.329**  **(<.001)** | **-.222**  **(.005)** | -.115  (.152) | **.557**  **(<.001)** | **.226**  **(.006)** | - |

Note. Bold characters indicate a significant result (*p*<0.05).

Table A3-b. Correlation matrix *(r_τ_, p)* of study variables in the sub-sample during follow-up care (multiply imputed dataset)

|  |  | 1 | 2 | 3 | 4 | 5 | 6 | 7 | 8 | 9 | 10 | 11 | 12 | 13 | 14 | 15 | 16 | 17 |
| --- | --- | --- | --- | --- | --- | --- | --- | --- | --- | --- | --- | --- | --- | --- | --- | --- | --- | --- |
| 1 | HRQoL | - |  |  |  |  |  |  |  |  |  |  |  |  |  |  |  |  |
| 2 | Child’s IPQ-R Symptoms | **-.254**  **(.021)** | - |  |  |  |  |  |  |  |  |  |  |  |  |  |  |  |
| 3 | Child’s IPQ-R Timeline-acute/chronic | .037  (.738) | -.196  (.079) | - |  |  |  |  |  |  |  |  |  |  |  |  |  |  |
| 4 | Child’s IPQ-R Timeline-cyclical | -.126  (.260) | .071  (.529) | .192  (.081) | - |  |  |  |  |  |  |  |  |  |  |  |  |  |
| 5 | Child’s IPQ-R Consequences | -.204  (.064) | .067  (.552) | .193  (.079) | **.243**  **(.026)** | - |  |  |  |  |  |  |  |  |  |  |  |  |
| 6 | Child’s IPQ-R Coherence | .046  (.677) | .027  (.809) | -.196  (.076) | **-.254**  **(.020)** | -.089  (.426) | - |  |  |  |  |  |  |  |  |  |  |  |
| 7 | Child’s IPQ-R Personal control | .195  (.077) | -.077  (.491) | .041  (.715) | .054  (.625) | .072  (.518) | -.049  (.665) | - |  |  |  |  |  |  |  |  |  |  |
| 8 | Child’s IPQ-R Emotional representations | -.095  (.391) | -.047  (.676) | .154  (.163) | **.232**  **(.034)** | **.251**  **(.021)** | -.143  (.195) | **.247**  **(.023)** | - |  |  |  |  |  |  |  |  |  |
| 9 | Parent’s IPQ-R Symptoms | -.150  (.186) | **.315**  **(.003)** | -.056  (.629) | .139  (.228) | .019  (.866) | .073  (.530) | .016  (.887) | -.039  (.732) | - |  |  |  |  |  |  |  |  |
| 10 | Parent’s IPQ-R Timeline-acute/chronic | -.053  (.633) | -.091  (.420) | **.397**  **(<.001)** | .053  (.635) | .092  (.410) | -.037  (.739) | -.023  (.837) | .156  (.161) | -.023  (.841) | - |  |  |  |  |  |  |  |
| 11 | Parent’s IPQ-R Timeline-cyclical | -.042  (.705) | .015  (.897) | .190  (.088) | **.275**  **(.011)** | .088  (.439) | -.196  (.077) | -.008  (.945) | .042  (.705) | .125  (.274) | .176  (.114) | - |  |  |  |  |  |  |
| 12 | Parent’s IPQ-R Consequences | **-.219**  **(.046)** | .008  (.941) | .069  (.537) | .128  (.252) | **.277**  **(.011)** | -.150  (.173) | -.010  (.931) | .042  (.707) | **.233**  **(.042)** | **.221**  **(.043)** | **.319**  **(.003)** | - |  |  |  |  |  |
| 13 | Parent’s IPQ-R Coherence | .077  (.486) | -.061  (.586) | -.076  (.494) | -.102  (.359) | -.060  (.588) | .128  (.248) | .005  (.961) | .026  (.815) | -.121  (.292) | .007  (.950) | .002  (.982) | -.157  (.154) | - |  |  |  |  |
| 14 | Parent’s IPQ-R Personal control | -.023  (.838) | .090  (.422) | -.016  (.889) | .094  (.404) | .136  (.224) | .046  (.681) | .144  (.193) | .015  (.891) | .103  (.355) | -.105  (.346) | .025  (.825) | .106  (.337) | .182  (.099) | - |  |  |  |
| 15 | Parent’s IPQ-R Emotional representations | -.035  (.753) | .046  (.677) | .092  (.407) | .084  (.449) | .148  (.180) | -.132  (.232) | -.049  (.664) | .062  (.580) | .074  (.508) | .108  (.331) | **.277**  **(.011)** | **.324**  **(.003)** | **-.343**  **(.001)** | -.150  (.178) | - |  |  |
| 16 | Child’ FoP | **-.319**  **(.004)** | .211  (.062) | .066  (.561) | .172  (.127) | .158  (.152) | -.071  (.521) | -.081  (.477) | **.226**  **(.044)** | .073  (.515) | .047  (.680) | .187  (.100) | .070  (.542) | -.042  (.711) | -.037  (.741) | .133  (.245) | - |  |
| 17 | Parent’s FoP | -.039  (.726) | .061  (.589) | .138  (.213) | .159  (.155) | .201  (.067) | -.215  (.050) | .055  (.621) | .167  (.129) | .076  (.510) | **.253**  **(.020)** | **.283**  **(.011)** | **.330**  **(.002)** | **-.224**  **(.041)** | -.109  (.328) | **.557**  **(<.001)** | .206  (.066) | - |

Note. Bold characters indicate a significant result (*p*<0.05)
