# Supplementary material for: Illness perceptions, fear of progression and health-related quality of life during acute treatment and follow-up care in paediatric cancer patients and their parents: a cross-sectional study
Source: BMC Psychol. 2023 Feb 13;11:44. doi: 10.1186/s40359-023-01078-6 (PMC9926758; doi:10.1186/s40359-023-01078-6)
Supplement: Supplementary file 5 — Additional file 5. Assumptions for a hierarchical multiple regression analysis for the follow-up care sample (n=84). [file 40359_2023_1078_MOESM5_ESM.docx]

## **Additional File 5:** Assumptions for a hierarchical multiple regression analysis for the follow-up care sample (*n*=84)

All assumptions for a hierarchical multiple regression analysis were met: (1) Residuals were normally distributed, as assessed by the Kolmogorov-Smirnov test, *p*=.200, the Shapiro-Wilk test, *p*=.731, and the visual exploration of the standardized residual’s histogram. (2) Residuals were homoscedastic, as assessed by the White test, *Χ^2^*(35)=30.939, *p*=.665. (3) Exploration of partial regression diagrams indicated linear associations between the independent variables and the dependent variable. (4) There was no evidence for multicollinearity, as assessed by the tolerance statistics / variance inflation factor and the condition indices^1^. (5) Outlier diagnostics detected no outliers, as shown by studentized deleted residuals ranging from {-2.6138; 2.1446} and Cook’s distances ranging from {.00001; .1997}. No leverage value was >.448 (formula *(3×predictors)/n* suggested by Velleman and Welsch^2^).

## References

1. Kim, J. H. Multicollinearity and misleading statistical results. *Korean J. Anesthesiol.* **72**, 558–569 (2019).

2. Velleman, P. F. & Welsch, R. E. Efficient Computing of Regression Diagnostics. *Am. Stat.* **35**, 234–242 (1981).
