# Supplementary material for: Illness perceptions, fear of progression and health-related quality of life during acute treatment and follow-up care in paediatric cancer patients and their parents: a cross-sectional study
Source: BMC Psychol. 2023 Feb 13;11:44. doi: 10.1186/s40359-023-01078-6 (PMC9926758; doi:10.1186/s40359-023-01078-6)
Supplement: Supplementary file 6 — Additional file 6. Hierarchical multiple regression analysis for the outcome child’s HRQoL in follow-up care, using the original dataset (n=68). [file 40359_2023_1078_MOESM6_ESM.docx]

## **Additional File 6:** Hierarchical multiple regression analysis for the outcome child’s HRQoL in follow-up care, using the original dataset (*n*=68)

| Predictor | *B* | SE *B* | *t* (*p*) | *R²* | *ΔR²* | *F* change (*p*) |
| --- | --- | --- | --- | --- | --- | --- |
| **Step 1: Sociodemographic and medical variables** |  |  |  | .202 | .202 | 3.143 (.014) |
| (Constant) | 98.464 | 7.539 | 13.060 (<.001) |  |  |  |
| Child’s Age | -1.356 | 0.569 | -2.384 (.020) |  |  |  |
| Diagnosis: lymphoma (dummy variable)^†^ | -0.911 | 4.442 | -.205 (.838) |  |  |  |
| Diagnosis: tumour of the central nervous system (dummy variable)^†^ | -4.418 | 3.489 | -1.266 (.210) |  |  |  |
| Diagnosis: other solid tumour (dummy variable)^†^ | -0.085 | 3.790 | -0.023 (.982) |  |  |  |
| Time since diagnosis | -0.060 | 0.034 | -1.769 (.082) |  |  |  |
| **Step 2: Child’s illness perceptions** |  |  |  | .274 | .072 | 6.023 (.017) |
| (Constant) | 99.343 | 7.260 | 13.684 (<.001) |  |  |  |
| Child’s Age | -0.893 | 0.578 | -1.544 (.128) |  |  |  |
| Diagnosis: lymphoma (dummy variable)^†^ | 0.336 | 4.302 | 0.078 (.938) |  |  |  |
| Diagnosis: tumour of the central nervous system (dummy variable)^†^ | -4.956 | 3.363 | -1.473 (.146) |  |  |  |
| Diagnosis: other solid tumour (dummy variable)^†^ | 0.277 | 3.648 | 0.076 (.940) |  |  |  |
| Time since diagnosis | -0.072 | 0.033 | -2.174 (.034) |  |  |  |
| Child’s IPQ-R Symptoms | -1.089 | 0.444 | -2.454 (.017) |  |  |  |
| **Step 3: Child’s FoP** |  |  |  | .368 | .094 | 8.938 (.004) |
| (Constant) | 105.269 | 7.111 | 14.803 (<.001) |  |  |  |
| Child’s Age | -0.748 | 0.546 | -1.369 (.176) |  |  |  |
| Diagnosis: lymphoma (dummy variable)^†^ | 1.643 | 4.071 | 0.404 (.688) |  |  |  |
| Diagnosis: tumour of the central nervous system (dummy variable)^†^ | -2.896 | 3.238 | -0.895 (.375) |  |  |  |
| Diagnosis: other solid tumour (dummy variable)^†^ | -0.309 | 3.437 | -0.090 (.929) |  |  |  |
| Time since diagnosis | -0.064 | 0.031 | -2.037 (.046) |  |  |  |
| Child’s IPQ-R Symptoms | -0.867 | 0.424 | -2.045 (.045) |  |  |  |
| Child’s FoP | -0.422 | 0.141 | -2.990 (.004) |  |  |  |
| **Step 4: Parent’s illness perceptions** |  |  |  | .430 | .062 | 6.401 (.014) |
| (Constant) | 116.671 | 8.167 | 14.286 (<.001) |  |  |  |
| Child’s Age | -0.887 | 0.526 | -1.686 (.097) |  |  |  |
| Diagnosis: lymphoma (dummy variable)^†^ | -0.267 | 3.971 | -0.067 (.947) |  |  |  |
| Diagnosis: tumour of the central nervous system (dummy variable)^†^ | -1.082 | 3.183 | -0.340 (.735) |  |  |  |
| Diagnosis: other solid tumour (dummy variable)^†^ | -0.112 | 3.293 | -0.034 (.973) |  |  |  |
| Time since diagnosis | -0.046 | 0.031 | -1.497 (.140) |  |  |  |
| Child’s IPQ-R Symptoms | -0.697 | 0.412 | -1.694 (.096) |  |  |  |
| Child’s FoP | -0.411 | 0.135 | -3.035 (.004) |  |  |  |
| Parent’s IPQ-R Consequences | -1.171 | 0.463 | -2.530 (.014) |  |  |  |

Note. ^†^ Reference category: Leukaemia.
